# (3aR*,6S*,7S*,7aR*)-2-(4-Methoxy­benz­yl)-7-(4-nitro­phen­yl)-6-phenyl-3a,6,7,7a-tetra­hydro­isoindolin-1-one

**DOI:** 10.1107/S1600536810017010

**Published:** 2010-05-19

**Authors:** Jian Zhao, Jin-Long Wu

**Affiliations:** a Laboratory of Asymmetric Catalysis and Synthesis, Department of Chemistry, Zhejiang University, Hangzhou, Zhejiang 310027, People’s Republic of China

## Abstract

The title compound, C_28_H_26_N_2_O_4_, crystallizes as a racemate with four stereogenic centers. In the molecule, the pyrrolidone ring adopts an envelope conformation and the cyclo­hexene ring has a twisted envelope conformation. In the crystal structure, mol­ecules are linked by weak inter­molecular C—H⋯O hydrogen bonds.

## Related literature

For bioactive compounds, see: Walling *et al.* (1988 ▶); Liu *et al.* (2006 ▶, 2008 ▶). For microwave-assisted intra­molecular Diels–Alder cyclo­addition, see: Wang *et al.* (2009 ▶); Wu *et al.* (2006 ▶, 2007 ▶). For the synthesis of title compound, see: Wu *et al.* (2009 ▶).
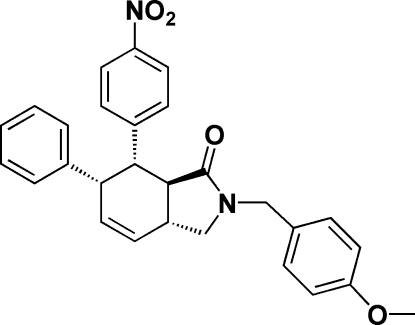


## Experimental

### 

#### Crystal data

C_28_H_26_N_2_O_4_
*M* *_r_* = 454.51
Triclinic, 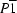

*a* = 5.4369 (4) Å
*b* = 12.2662 (7) Å
*c* = 18.149 (1) Å
α = 79.633 (1)°
β = 84.036 (2)°
γ = 80.325 (2)°
*V* = 1170.25 (13) Å^3^
*Z* = 2
Mo *K*α radiation
μ = 0.09 mm^−1^
*T* = 296 K
0.37 × 0.31 × 0.18 mm

#### Data collection

Rigaku R-AXIS RAPID diffractometer
Absorption correction: multi-scan (*ABSCOR*; Higashi, 1995 ▶) *T*_min_ = 0.967, *T*_max_ = 0.985
11469 measured reflections
5285 independent reflections
3402 reflections with *I* > 2σ(*I*)
*R*_int_ = 0.024

#### Refinement

*R*[*F* ^2^ > 2σ(*F* ^2^)] = 0.042
*wR*(*F* ^2^) = 0.123
*S* = 1.01
5285 reflections
309 parameters
H-atom parameters constrained
Δρ_max_ = 0.23 e Å^−3^
Δρ_min_ = −0.22 e Å^−3^

### 

Data collection: *PROCESS-AUTO* (Rigaku, 2006 ▶); cell refinement: *PROCESS-AUTO*; data reduction: *CrystalStructure* (Rigaku, 2007 ▶); program(s) used to solve structure: *SHELXS97* (Sheldrick, 2008 ▶); program(s) used to refine structure: *SHELXL97* (Sheldrick, 2008 ▶); molecular graphics: *ORTEP-3* (Farrugia, 1997 ▶); software used to prepare material for publication: *WinGX* (Farrugia, 1999 ▶).

## Supplementary Material

Crystal structure: contains datablocks I, global. DOI: 10.1107/S1600536810017010/lx2150sup1.cif

Structure factors: contains datablocks I. DOI: 10.1107/S1600536810017010/lx2150Isup2.hkl

Additional supplementary materials:  crystallographic information; 3D view; checkCIF report

## Figures and Tables

**Table 1:** Hydrogen-bond geometry (Å, °)

*D*—H⋯*A*	*D*—H	H⋯*A*	*D*⋯*A*	*D*—H⋯*A*
C8—H8*A*⋯O3^i^	0.97	2.63	3.239 (2)	122
C28—H28*A*⋯O1^ii^	0.96	2.55	3.242 (2)	129

Symmetry codes: (i) 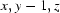; (ii) 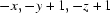.

**Fig. 1.**
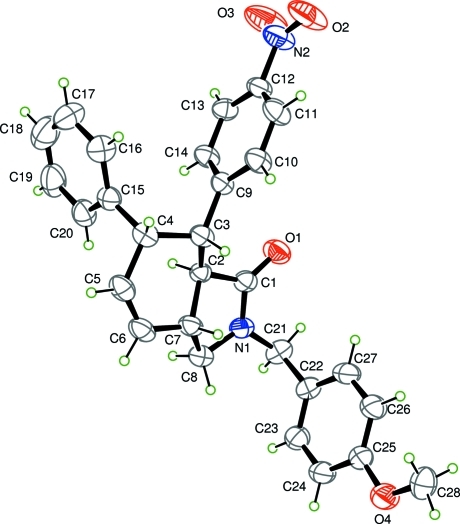
The molecular structure of the title compound with the atom numbering scheme. Displacement ellipsoids are drawn at 50% probability level. H atoms are presented as a small spheres of arbitrary radius.

**Fig. 2.**
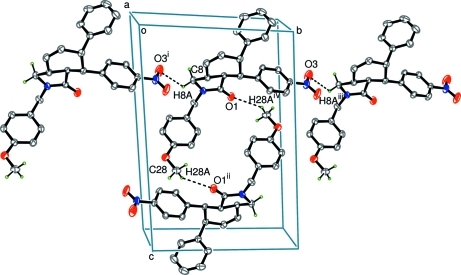
C–H···O interactions (dotted lines) in the crystal structure of the title compound. [Symmetry codes: (i) x, y - 1, z; (ii) - x, - y + 1, - z + 1 ; (iii) x, y + 1, z; (iv) - x, - y + 1, - z + 1. ]

## supplementary crystallographic information

### Comment

The title compound, C_28_H_26_N_2_O_4_, has a hexahydro-1*H*-isoindolone core, which is present in both synthetic and naturally occurring bioactive compounds (Walling *et al.*, 1988 and Liu *et al.*, 2006, 2008). The title compound has recently been obtained during microwave-assisted intramolecular Diels-Alder cycloaddition along with a minor diastereomer with a 82:18 diastereomeric ratio (Wang *et al.*, 2009; Wu *et al*., 2006, 2007, 2009). The compound has four stereogenic centers but crystallizes as a racemate as indicated by the centrosymmetric space group. Here we report the crystal structure of the title compound (Fig. 1).

In the crystal structure of the title compound, there are one pyrrolidone ring and one cyclohexene ring. The pyrrolidone ring C1-C2/C7-C8/N1 adopts envelope conformation, whereas the cyclohexene ring C2-C7 has a twisted envelope conformation. Bond length of C3–C4 is larger than normal C–C single bond because of the hindrance between two phenyl rings at C3 and C4.

The crystal packing (Fig. 2) is stabilized by weak non-classical intermolecular C–H···O hydrogen bonds; the first one between the pyrrolidone H atom and the oxygen of the nitro group, with a C8–H8A···O3^i^, and the second one between an H atom of the methoxy group and the oxygen of the C═O unit, with a C28–H28A···O1^ii^, respectively (Table 1).

### Experimental

To a 10-mL pressurized process vial was added *N*-(4-methoxybenzyl)- *N*-(2*E*,4*E*)-5-phenylpenta-2,4-dienyl 2-bromoacetamide (133.0 mg, 0.33 mmol), triphenylphosphine (104.0 mg, 0.40 mmol), K_2_CO_3_ (68.0 mg, 0.50 mmol), and 4-nitrobenzaldehyde (60.0 mg, 0.40 mmol). After adding aqueous THF (H_2_O:THF = 1:1, 3.5 mL) the loaded vial was then sealed with a cap containing a silicon septum followed by stirring the mixture at room temperature for 6 h to allow the Wittig olefination taking place among the 2-bromoacetamide and the aldehyde. The vial containing the crude Wittig product was then put into the cavity of a technical microwave reactor with the temperature measured by an IR sensor. After heating at 453 K for 0.5 h, the reaction mixture was successively washed with saturated aqueous NH_4_Cl and brine, and then extracted with EtOAc (3 x 5 mL). The combined organic layer was dried over Na_2_SO_4_, filtered, and evaporated under reduced pressure. The residue was purified by flash column chromatography (silica gel, 20% EtOAc in petroleum ether) to furnish the title compound (82.0 mg, 55%), along with a minor diastereomer (17.9 mg, 12%), as a colorless solid. mp 466-468 K (CH_2_Cl_2_-EtOAc-hexane). Single crystals, as a racemate, suitable for X-ray diffraction of the title compound were grown at ambient temperature in the mixed solvent of methylene chloride, ethyl acetate and hexane (v:v:v = 1:1:3).

### Refinement

The H atoms were placed in calculated positions with C–H = 0.93-0.98 Å, and included in the refinement in riding model, with *U*_iso_(H) = 1.2*U*_eq_ (carrier atom).

### Crystal data

**Table:**

C_28_H_26_N_2_O_4_	*Z* = 2
*M**_r_* = 454.51	*F*(000) = 480
Triclinic, *P*1	*D*_x_ = 1.290 Mg m^−^^3^
Hall symbol: -P 1	Mo *K*α radiation, λ = 0.71073 Å
*a* = 5.4369 (4) Å	Cell parameters from 7298 reflections
*b* = 12.2662 (7) Å	θ = 3.1–27.4°
*c* = 18.149 (1) Å	µ = 0.09 mm^−^^1^
α = 79.633 (1)°	*T* = 296 K
β = 84.036 (2)°	Block, colorless
γ = 80.325 (2)°	0.37 × 0.31 × 0.18 mm
*V* = 1170.25 (13) Å^3^	

### Data collection

**Table:**

Rigaku R-AXIS RAPID diffractometer	5285 independent reflections
Radiation source: rotating anode	3402 reflections with *I* > 2σ(*I*)
graphite	*R*_int_ = 0.024
Detector resolution: 10.00 pixels mm^-1^	θ_max_ = 27.4°, θ_min_ = 3.1°
ω scans	*h* = −7→7
Absorption correction: multi-scan (*ABSCOR*; Higashi, 1995)	*k* = −15→15
*T*_min_ = 0.967, *T*_max_ = 0.985	*l* = −23→23
11469 measured reflections	

### Refinement

**Table:**

Refinement on *F*^2^	Secondary atom site location: difference Fourier map
Least-squares matrix: full	Hydrogen site location: difference Fourier map
*R*[*F*^2^ > 2σ(*F*^2^)] = 0.042	H-atom parameters constrained
*wR*(*F*^2^) = 0.123	*w* = 1/[σ^2^(*F*_o_^2^) + (0.041*P*)^2^ + 0.4229*P*] where *P* = (*F*_o_^2^ + 2*F*_c_^2^)/3
*S* = 1.01	(Δ/σ)_max_ < 0.001
5285 reflections	Δρ_max_ = 0.23 e Å^−^^3^
309 parameters	Δρ_min_ = −0.22 e Å^−^^3^
0 restraints	Extinction correction: *SHELXL97* (Sheldrick, 2008), Fc*=kFc[1+0.001xFc^2^λ^3^/sin(2θ)]^-1/4^
Primary atom site location: structure-invariant direct methods	Extinction coefficient: 0.030 (2)

### Special details

**Table:**

Geometry. All esds (except the esd in the dihedral angle between two l.s. planes) are estimated using the full covariance matrix. The cell esds are taken into account individually in the estimation of esds in distances, angles and torsion angles; correlations between esds in cell parameters are only used when they are defined by crystal symmetry. An approximate (isotropic) treatment of cell esds is used for estimating esds involving l.s. planes.
Refinement. Refinement of *F*^2^ against ALL reflections. The weighted *R*-factor wR and goodness of fit *S* are based on *F*^2^, conventional *R*-factors *R* are based on *F*, with *F* set to zero for negative *F*^2^. The threshold expression of *F*^2^ > σ(*F*^2^) is used only for calculating *R*-factors(gt) etc. and is not relevant to the choice of reflections for refinement. *R*-factors based on *F*^2^ are statistically about twice as large as those based on *F*, and *R*- factors based on ALL data will be even larger.

### Fractional atomic coordinates and isotropic or equivalent isotropic displacement parameters (Å^2^)

**Table:**

	*x*	*y*	*z*	*U*_iso_*/*U*_eq_	
O1	0.3920 (3)	0.60330 (10)	0.32284 (8)	0.0562 (4)	
C3	−0.0091 (3)	0.63563 (13)	0.20272 (9)	0.0372 (4)	
H3	−0.1434	0.6128	0.2396	0.045*	
C9	0.0145 (3)	0.75414 (13)	0.21103 (9)	0.0354 (4)	
O4	−0.0827 (3)	0.11519 (11)	0.56486 (8)	0.0626 (4)	
C10	−0.1747 (3)	0.81473 (14)	0.25156 (10)	0.0437 (4)	
H10	−0.3107	0.7810	0.2743	0.052*	
C4	−0.0937 (3)	0.62877 (14)	0.12304 (10)	0.0403 (4)	
H4	−0.2705	0.6622	0.1227	0.048*	
C2	0.2183 (3)	0.54638 (12)	0.21746 (9)	0.0345 (4)	
H2	0.3394	0.5560	0.1738	0.041*	
N1	0.4524 (3)	0.42079 (11)	0.30303 (8)	0.0451 (4)	
C13	0.2321 (3)	0.91472 (14)	0.18609 (10)	0.0422 (4)	
H13	0.3688	0.9486	0.1643	0.051*	
C14	0.2179 (3)	0.80600 (13)	0.17894 (10)	0.0410 (4)	
H14	0.3471	0.7665	0.1521	0.049*	
C25	0.0719 (4)	0.18191 (15)	0.52078 (10)	0.0463 (4)	
C11	−0.1642 (4)	0.92434 (15)	0.25872 (11)	0.0510 (5)	
H11	−0.2930	0.9648	0.2851	0.061*	
C12	0.0403 (3)	0.97220 (13)	0.22604 (10)	0.0429 (4)	
C15	0.0421 (3)	0.69472 (14)	0.05692 (10)	0.0417 (4)	
C1	0.3594 (3)	0.53126 (13)	0.28773 (9)	0.0391 (4)	
C26	0.0549 (4)	0.29610 (15)	0.51910 (11)	0.0532 (5)	
H26	−0.0676	0.3333	0.5494	0.064*	
C5	−0.0810 (3)	0.50875 (15)	0.11146 (11)	0.0454 (4)	
H5	−0.1559	0.4971	0.0703	0.054*	
C7	0.1409 (3)	0.43048 (13)	0.22418 (10)	0.0415 (4)	
H7	0.0091	0.4250	0.2652	0.050*	
C22	0.4095 (3)	0.30153 (14)	0.42622 (10)	0.0444 (4)	
C6	0.0281 (3)	0.41877 (15)	0.15518 (11)	0.0473 (4)	
H6	0.0342	0.3481	0.1427	0.057*	
N2	0.0562 (4)	1.08752 (13)	0.23437 (11)	0.0613 (5)	
C27	0.2232 (4)	0.35445 (15)	0.47165 (11)	0.0531 (5)	
H27	0.2107	0.4313	0.4703	0.064*	
C24	0.2572 (4)	0.12738 (15)	0.47615 (11)	0.0541 (5)	
H24	0.2693	0.0505	0.4774	0.065*	
O2	−0.1207 (4)	1.14155 (13)	0.26491 (12)	0.0936 (6)	
C23	0.4236 (4)	0.18681 (15)	0.42990 (11)	0.0518 (5)	
H23	0.5482	0.1491	0.4005	0.062*	
C8	0.3733 (4)	0.35368 (14)	0.25297 (11)	0.0486 (5)	
H8A	0.3336	0.2824	0.2802	0.058*	
H8B	0.4999	0.3405	0.2124	0.058*	
O3	0.2473 (4)	1.12510 (13)	0.20977 (13)	0.0982 (7)	
C20	0.2721 (4)	0.65034 (17)	0.02460 (10)	0.0491 (4)	
H20	0.3444	0.5777	0.0433	0.059*	
C21	0.5791 (4)	0.36794 (16)	0.37105 (11)	0.0507 (5)	
H21A	0.6282	0.4253	0.3944	0.061*	
H21B	0.7294	0.3185	0.3575	0.061*	
C16	−0.0606 (4)	0.80281 (17)	0.02643 (12)	0.0608 (5)	
H16	−0.2147	0.8347	0.0465	0.073*	
C28	−0.2937 (4)	0.16809 (19)	0.60542 (12)	0.0601 (5)	
H28A	−0.2378	0.2032	0.6425	0.090*	
H28B	−0.3949	0.1130	0.6297	0.090*	
H28C	−0.3906	0.2238	0.5714	0.090*	
C19	0.3949 (5)	0.7124 (2)	−0.03484 (12)	0.0679 (6)	
H19	0.5496	0.6816	−0.0551	0.081*	
C17	0.0632 (6)	0.8639 (2)	−0.03356 (14)	0.0801 (8)	
H17	−0.0087	0.9362	−0.0533	0.096*	
C18	0.2906 (6)	0.8187 (2)	−0.06392 (14)	0.0814 (8)	
H18	0.3733	0.8601	−0.1040	0.098*	

### Atomic displacement parameters (Å^2^)

**Table:**

	*U*^11^	*U*^22^	*U*^33^	*U*^12^	*U*^13^	*U*^23^
O1	0.0704 (9)	0.0431 (7)	0.0610 (8)	−0.0104 (6)	−0.0178 (7)	−0.0151 (6)
C3	0.0321 (9)	0.0353 (8)	0.0455 (9)	−0.0097 (7)	0.0068 (7)	−0.0113 (7)
C9	0.0323 (9)	0.0350 (8)	0.0391 (9)	−0.0052 (6)	0.0006 (7)	−0.0089 (7)
O4	0.0693 (10)	0.0519 (8)	0.0665 (9)	−0.0207 (7)	0.0211 (7)	−0.0142 (7)
C10	0.0363 (9)	0.0427 (9)	0.0534 (10)	−0.0074 (7)	0.0062 (8)	−0.0157 (8)
C4	0.0284 (9)	0.0415 (9)	0.0527 (10)	−0.0055 (7)	−0.0021 (7)	−0.0126 (8)
C2	0.0352 (9)	0.0305 (8)	0.0378 (8)	−0.0085 (6)	0.0042 (7)	−0.0062 (7)
N1	0.0567 (10)	0.0342 (7)	0.0440 (8)	−0.0094 (7)	−0.0054 (7)	−0.0021 (6)
C13	0.0399 (10)	0.0366 (8)	0.0505 (10)	−0.0101 (7)	−0.0004 (8)	−0.0058 (8)
C14	0.0325 (9)	0.0373 (8)	0.0536 (10)	−0.0056 (7)	0.0066 (7)	−0.0143 (8)
C25	0.0514 (11)	0.0427 (9)	0.0447 (10)	−0.0115 (8)	0.0029 (8)	−0.0067 (8)
C11	0.0441 (11)	0.0470 (10)	0.0634 (12)	−0.0027 (8)	0.0085 (9)	−0.0240 (9)
C12	0.0482 (11)	0.0302 (8)	0.0516 (10)	−0.0028 (7)	−0.0070 (8)	−0.0116 (8)
C15	0.0423 (10)	0.0425 (9)	0.0439 (9)	−0.0097 (7)	−0.0091 (8)	−0.0105 (8)
C1	0.0404 (10)	0.0347 (8)	0.0430 (9)	−0.0122 (7)	0.0042 (7)	−0.0065 (7)
C26	0.0549 (12)	0.0434 (10)	0.0580 (12)	−0.0023 (9)	0.0089 (9)	−0.0120 (9)
C5	0.0419 (10)	0.0475 (10)	0.0535 (10)	−0.0161 (8)	−0.0042 (8)	−0.0175 (9)
C7	0.0470 (10)	0.0330 (8)	0.0467 (10)	−0.0144 (7)	0.0051 (8)	−0.0093 (7)
C22	0.0454 (11)	0.0403 (9)	0.0446 (10)	−0.0052 (8)	−0.0064 (8)	0.0014 (8)
C6	0.0497 (11)	0.0393 (9)	0.0590 (11)	−0.0180 (8)	0.0013 (9)	−0.0175 (9)
N2	0.0682 (12)	0.0380 (8)	0.0807 (13)	−0.0074 (8)	−0.0033 (10)	−0.0200 (9)
C27	0.0619 (13)	0.0339 (9)	0.0602 (12)	−0.0049 (8)	0.0016 (10)	−0.0055 (9)
C24	0.0660 (13)	0.0351 (9)	0.0590 (12)	−0.0079 (9)	0.0096 (10)	−0.0097 (9)
O2	0.0970 (14)	0.0506 (9)	0.1350 (16)	−0.0010 (9)	0.0198 (12)	−0.0457 (10)
C23	0.0573 (12)	0.0421 (10)	0.0519 (11)	−0.0028 (8)	0.0080 (9)	−0.0081 (9)
C8	0.0625 (12)	0.0324 (8)	0.0509 (11)	−0.0080 (8)	−0.0036 (9)	−0.0069 (8)
O3	0.0893 (13)	0.0558 (9)	0.1607 (19)	−0.0331 (9)	0.0210 (13)	−0.0445 (11)
C20	0.0476 (11)	0.0564 (11)	0.0470 (10)	−0.0139 (9)	−0.0005 (8)	−0.0146 (9)
C21	0.0492 (11)	0.0485 (10)	0.0512 (11)	−0.0102 (8)	−0.0058 (9)	0.0035 (9)
C16	0.0701 (14)	0.0524 (11)	0.0585 (12)	−0.0043 (10)	−0.0128 (11)	−0.0055 (10)
C28	0.0536 (13)	0.0750 (14)	0.0512 (12)	−0.0146 (10)	0.0070 (10)	−0.0106 (10)
C19	0.0690 (15)	0.0910 (17)	0.0500 (12)	−0.0321 (13)	0.0083 (11)	−0.0174 (12)
C17	0.121 (2)	0.0580 (13)	0.0596 (14)	−0.0195 (14)	−0.0225 (15)	0.0097 (11)
C18	0.106 (2)	0.0889 (18)	0.0527 (14)	−0.0455 (17)	−0.0008 (14)	0.0034 (13)

### Geometric parameters (Å, °)

**Table:**

O1—C1	1.2235 (19)	C26—H26	0.9300
C3—C9	1.516 (2)	C5—C6	1.328 (3)
C3—C2	1.519 (2)	C5—H5	0.9300
C3—C4	1.583 (2)	C7—C6	1.489 (2)
C3—H3	0.9800	C7—C8	1.522 (3)
C9—C10	1.391 (2)	C7—H7	0.9800
C9—C14	1.392 (2)	C22—C23	1.386 (2)
O4—C25	1.368 (2)	C22—C27	1.388 (3)
O4—C28	1.423 (2)	C22—C21	1.511 (2)
C10—C11	1.385 (2)	C6—H6	0.9300
C10—H10	0.9300	N2—O2	1.216 (2)
C4—C5	1.513 (2)	N2—O3	1.217 (2)
C4—C15	1.519 (2)	C27—H27	0.9300
C4—H4	0.9800	C24—C23	1.378 (3)
C2—C1	1.523 (2)	C24—H24	0.9300
C2—C7	1.530 (2)	C23—H23	0.9300
C2—H2	0.9800	C8—H8A	0.9700
N1—C1	1.354 (2)	C8—H8B	0.9700
N1—C8	1.469 (2)	C20—C19	1.384 (3)
N1—C21	1.469 (2)	C20—H20	0.9300
C13—C12	1.375 (2)	C21—H21A	0.9700
C13—C14	1.378 (2)	C21—H21B	0.9700
C13—H13	0.9300	C16—C17	1.387 (3)
C14—H14	0.9300	C16—H16	0.9300
C25—C26	1.383 (2)	C28—H28A	0.9600
C25—C24	1.385 (3)	C28—H28B	0.9600
C11—C12	1.375 (2)	C28—H28C	0.9600
C11—H11	0.9300	C19—C18	1.366 (4)
C12—N2	1.467 (2)	C19—H19	0.9300
C15—C16	1.388 (3)	C17—C18	1.370 (4)
C15—C20	1.392 (3)	C17—H17	0.9300
C26—C27	1.387 (3)	C18—H18	0.9300
			
C9—C3—C2	117.16 (13)	C6—C7—C2	111.36 (14)
C9—C3—C4	112.57 (13)	C8—C7—C2	101.77 (13)
C2—C3—C4	107.27 (12)	C6—C7—H7	106.7
C9—C3—H3	106.4	C8—C7—H7	106.7
C2—C3—H3	106.4	C2—C7—H7	106.7
C4—C3—H3	106.4	C23—C22—C27	117.55 (17)
C10—C9—C14	118.12 (15)	C23—C22—C21	121.30 (17)
C10—C9—C3	119.26 (14)	C27—C22—C21	121.02 (16)
C14—C9—C3	122.61 (14)	C5—C6—C7	120.34 (15)
C25—O4—C28	117.97 (15)	C5—C6—H6	119.8
C11—C10—C9	121.17 (16)	C7—C6—H6	119.8
C11—C10—H10	119.4	O2—N2—O3	122.85 (17)
C9—C10—H10	119.4	O2—N2—C12	119.12 (18)
C5—C4—C15	110.34 (14)	O3—N2—C12	118.03 (17)
C5—C4—C3	111.94 (14)	C26—C27—C22	121.97 (17)
C15—C4—C3	114.83 (13)	C26—C27—H27	119.0
C5—C4—H4	106.4	C22—C27—H27	119.0
C15—C4—H4	106.4	C23—C24—C25	120.09 (17)
C3—C4—H4	106.4	C23—C24—H24	120.0
C3—C2—C1	122.36 (13)	C25—C24—H24	120.0
C3—C2—C7	109.16 (13)	C24—C23—C22	121.43 (17)
C1—C2—C7	101.14 (13)	C24—C23—H23	119.3
C3—C2—H2	107.8	C22—C23—H23	119.3
C1—C2—H2	107.8	N1—C8—C7	100.71 (13)
C7—C2—H2	107.8	N1—C8—H8A	111.6
C1—N1—C8	113.54 (14)	C7—C8—H8A	111.6
C1—N1—C21	123.90 (15)	N1—C8—H8B	111.6
C8—N1—C21	121.57 (14)	C7—C8—H8B	111.6
C12—C13—C14	118.76 (16)	H8A—C8—H8B	109.4
C12—C13—H13	120.6	C19—C20—C15	121.2 (2)
C14—C13—H13	120.6	C19—C20—H20	119.4
C13—C14—C9	121.40 (16)	C15—C20—H20	119.4
C13—C14—H14	119.3	N1—C21—C22	110.87 (15)
C9—C14—H14	119.3	N1—C21—H21A	109.5
O4—C25—C26	124.70 (17)	C22—C21—H21A	109.5
O4—C25—C24	115.44 (16)	N1—C21—H21B	109.5
C26—C25—C24	119.85 (17)	C22—C21—H21B	109.5
C12—C11—C10	118.70 (16)	H21A—C21—H21B	108.1
C12—C11—H11	120.7	C17—C16—C15	120.9 (2)
C10—C11—H11	120.7	C17—C16—H16	119.5
C13—C12—C11	121.85 (15)	C15—C16—H16	119.5
C13—C12—N2	118.85 (16)	O4—C28—H28A	109.5
C11—C12—N2	119.30 (16)	O4—C28—H28B	109.5
C16—C15—C20	117.41 (18)	H28A—C28—H28B	109.5
C16—C15—C4	120.60 (17)	O4—C28—H28C	109.5
C20—C15—C4	121.99 (16)	H28A—C28—H28C	109.5
O1—C1—N1	125.80 (17)	H28B—C28—H28C	109.5
O1—C1—C2	128.05 (15)	C18—C19—C20	120.5 (2)
N1—C1—C2	106.10 (13)	C18—C19—H19	119.8
C25—C26—C27	119.10 (17)	C20—C19—H19	119.8
C25—C26—H26	120.5	C18—C17—C16	120.5 (2)
C27—C26—H26	120.5	C18—C17—H17	119.7
C6—C5—C4	125.40 (16)	C16—C17—H17	119.7
C6—C5—H5	117.3	C19—C18—C17	119.5 (2)
C4—C5—H5	117.3	C19—C18—H18	120.2
C6—C7—C8	122.74 (15)	C17—C18—H18	120.2
			
C2—C3—C9—C10	−132.93 (17)	C15—C4—C5—C6	117.7 (2)
C4—C3—C9—C10	102.00 (18)	C3—C4—C5—C6	−11.5 (2)
C2—C3—C9—C14	47.7 (2)	C3—C2—C7—C6	57.77 (18)
C4—C3—C9—C14	−77.3 (2)	C1—C2—C7—C6	−172.00 (14)
C14—C9—C10—C11	1.3 (3)	C3—C2—C7—C8	−169.81 (14)
C3—C9—C10—C11	−178.11 (17)	C1—C2—C7—C8	−39.58 (16)
C9—C3—C4—C5	171.91 (14)	C4—C5—C6—C7	3.0 (3)
C2—C3—C4—C5	41.60 (17)	C8—C7—C6—C5	−146.39 (18)
C9—C3—C4—C15	45.10 (19)	C2—C7—C6—C5	−25.6 (2)
C2—C3—C4—C15	−85.20 (16)	C13—C12—N2—O2	174.7 (2)
C9—C3—C2—C1	49.5 (2)	C11—C12—N2—O2	−5.7 (3)
C4—C3—C2—C1	177.21 (13)	C13—C12—N2—O3	−4.9 (3)
C9—C3—C2—C7	167.06 (14)	C11—C12—N2—O3	174.6 (2)
C4—C3—C2—C7	−65.26 (16)	C25—C26—C27—C22	−0.5 (3)
C12—C13—C14—C9	0.2 (3)	C23—C22—C27—C26	−0.3 (3)
C10—C9—C14—C13	−0.7 (3)	C21—C22—C27—C26	175.68 (18)
C3—C9—C14—C13	178.63 (16)	O4—C25—C24—C23	−179.69 (18)
C28—O4—C25—C26	7.7 (3)	C26—C25—C24—C23	−0.3 (3)
C28—O4—C25—C24	−172.98 (18)	C25—C24—C23—C22	−0.6 (3)
C9—C10—C11—C12	−1.2 (3)	C27—C22—C23—C24	0.8 (3)
C14—C13—C12—C11	−0.1 (3)	C21—C22—C23—C24	−175.12 (18)
C14—C13—C12—N2	179.39 (17)	C1—N1—C8—C7	−20.81 (19)
C10—C11—C12—C13	0.7 (3)	C21—N1—C8—C7	148.23 (16)
C10—C11—C12—N2	−178.85 (17)	C6—C7—C8—N1	161.81 (15)
C5—C4—C15—C16	137.20 (17)	C2—C7—C8—N1	36.61 (16)
C3—C4—C15—C16	−95.2 (2)	C16—C15—C20—C19	1.0 (3)
C5—C4—C15—C20	−42.9 (2)	C4—C15—C20—C19	−178.91 (16)
C3—C4—C15—C20	84.73 (19)	C1—N1—C21—C22	104.26 (19)
C8—N1—C1—O1	177.63 (17)	C8—N1—C21—C22	−63.6 (2)
C21—N1—C1—O1	8.9 (3)	C23—C22—C21—N1	96.9 (2)
C8—N1—C1—C2	−4.52 (19)	C27—C22—C21—N1	−79.0 (2)
C21—N1—C1—C2	−173.27 (15)	C20—C15—C16—C17	−0.5 (3)
C3—C2—C1—O1	−33.0 (3)	C4—C15—C16—C17	179.46 (18)
C7—C2—C1—O1	−154.41 (18)	C15—C20—C19—C18	−1.0 (3)
C3—C2—C1—N1	149.18 (14)	C15—C16—C17—C18	−0.2 (4)
C7—C2—C1—N1	27.81 (16)	C20—C19—C18—C17	0.3 (4)
O4—C25—C26—C27	−179.84 (19)	C16—C17—C18—C19	0.2 (4)
C24—C25—C26—C27	0.8 (3)		

### Hydrogen-bond geometry (Å, °)

**Table:**

*D*—H···*A*	*D*—H	H···*A*	*D*···*A*	*D*—H···*A*
C8—H8A···O3^i^	0.97	2.63	3.239 (2)	122
C28—H28A···O1^ii^	0.96	2.55	3.242 (2)	129

Symmetry codes: (i) *x*, *y*−1, *z*; (ii) −*x*, −*y*+1, −*z*+1.
